# Calcined Bovine-Bone-Derived Ca–P as a Densification Filler for Tungsten/PVC Lead-Free Flexible X-Ray Shielding Sheets

**DOI:** 10.3390/polym18151858

**Published:** 2026-07-29

**Authors:** Seon-Chil Kim

**Affiliations:** Department of Biomedical Engineering, School of Medicine, Keimyung University, 1095 Dal-gubeol-daero, Daegu 42601, Republic of Korea; chil@kmu.ac.kr; Tel.: +82-10-4803-7773

**Keywords:** tungsten, calcination, Ca–P, medical radiation, shielding, radiation protection efficiency (RPE)

## Abstract

With the increasing use of diagnostic X-ray examinations and interventional procedures in medical institutions, the development of lead-free flexible X-ray shielding materials to reduce occupational exposure to scattered radiation has become increasingly important. Tungsten (W) is considered one of the most promising alternatives to lead (Pb) because of its high density and excellent attenuation characteristics. However, in highly filled composites, particle agglomeration and the formation of microvoids can reduce the effective density of the material, thereby limiting its shielding performance. In this study, calcined Ca–P inorganic powder derived from waste animal bone was applied as an auxiliary filler in W/PVC composite sheets to evaluate its potential for improving shielding performance through void reduction and effective density enhancement. Waste animal bone was calcined (600–1200 °C) to produce Ca–P powder. The calcined Ca–P was melt-compounded with tungsten/PVC and processed into 0.25 mm thick flexible shielding sheets. The fabricated sheets were characterized by cross-sectional scanning electron microscopy (SEM), bulk density measurements, and radiation protection efficiency (RPE) evaluations under effective X-ray energies ranging from 23.6 to 52.4 keV. SEM observations revealed a reduction in microvoids and the formation of a more continuous internal structure in the sheets containing calcined Ca–P. At the W-85 composition, the bulk density increased from 12.448 to 15.241 g/cm^3^, accompanied by an RPE improvement of up to 4.5 percentage points at 23.6 keV. Correspondingly, shielding performance improved by up to approximately 4.5 percentage points at 23.6 keV and by approximately 1.0–3.4 percentage points at 46.5 keV. These findings suggest that calcined Ca–P functions not as a primary shielding material replacing tungsten, but rather as a density-correcting auxiliary filler that mitigates microvoid formation and enhances the effective density of highly filled W/PVC composites. These findings demonstrate that waste animal bone-derived Ca–P is a promising upcycled densification filler for improving the processability and X-ray shielding performance of lead-free flexible shielding sheets.

## 1. Introduction

The increasing use of diagnostic X-ray examinations and radiation-guided interventional procedures in medical institutions highlights the importance of managing occupational exposure to scattered radiation in healthcare workers [1]. Various types of shielding products, including shielding curtains, mobile shielding barriers, aprons, thyroid protectors, and wearable shielding sheets, are widely used in clinical settings. These radiation shielding materials must simultaneously satisfy attenuation performance, flexibility, durability, convenience of wearing and handling, and economic feasibility [2,3,4]. Owing to their weight and thickness during prolonged use, wearable shielding products are directly associated with user fatigue and work efficiency; therefore, material designs that minimize an increase in weight while maintaining optimal shielding performance are desired [5,6].

Recently, concerns regarding toxicity, environmental regulations, and disposal burden associated with lead-based shielding materials have accelerated the transition toward lead-free alternatives, particularly environmentally friendly radiation-shielding materials [7]. However, materials that can simultaneously satisfy economic feasibility and processability comparable to lead have not yet been fully achieved, resulting in a growing interest in composite materials comprising various material combinations [8].

As an alternative lead-free shielding material, tungsten is a metal with a high density (19.3 g/cm^3^) that exhibits excellent attenuation efficiency through photoelectric absorption and scattering interactions in the X-ray energy range [9]. However, tungsten-based flexible shielding sheets have several structural limitations in practical applications. First, achieving the target shielding performance requires an extremely high filler loading, thereby increasing the costs of raw materials. Second, high-density metal loading increases the areal density of the sheet, potentially causing wearer fatigue and sagging owing to excessive weight [10]. Third, under high-loading conditions, particle agglomeration and non-uniform dispersion are likely to occur. The formation of micropores during mixing and sheet fabrication can reduce the density of the composite, thereby decreasing the actual shielding performance compared to that expected from the same composition [11]. Thus, the performance of tungsten-based shielding sheets is influenced by both the amount of shielding material and the filler loading ratio and is strongly affected by microstructural factors such as agglomeration, porosity, and effective density [12,13].

The fundamental principles of radiation attenuation can explain the interrelationships among microporosity, effective density, and shielding performance [14]. As X-rays pass through a material, their intensity decreases exponentially with thickness. This behavior is theoretically described by the linear attenuation law, which serves as the basis for evaluating the shielding performance [15]. Even for the same material, attenuation characteristics may vary depending on density. In particular, for mixtures or composite materials, the mass attenuation coefficient (μ/ρ) is more appropriate than the linear attenuation coefficient (μ). Notably, the mass attenuation coefficient of a mixture can be calculated using the mixture rule by weighing the mass attenuation coefficients of individual components according to their mass fractions [16].

In highly filled composite shielding sheets, micropores generated during the fabrication process are structural defects directly associated with density reduction within the sheet, resulting in deterioration of the shielding performance even at the same filler loading [17]. To improve the performance of tungsten-based shielding sheets, strategies focused solely on incorporating high atomic number materials may be insufficient. Instead, densification process technologies that reduce porosity and enhance the effective density of the composite through process optimization and microstructural control can provide an important approach for improving shielding performance.

In previous studies on lead-free X-ray shielding composites, various auxiliary fillers, including BaSO_4_, Bi_2_O_3_, and Gd_2_O_3_, have been incorporated alongside tungsten to compensate for the reduced processability and non-uniform particle dispersion associated with highly loaded tungsten systems, while simultaneously improving manufacturing feasibility and economic efficiency [18]. In addition to conventional inorganic auxiliary fillers, recent studies have increasingly investigated bio-derived materials as sustainable additives for improving the microstructure of composite materials [19,20]. Among these materials, animal bone-derived fillers have attracted increasing attention because of their high calcium phosphate content, natural abundance, and biocompatibility [21,22]. These studies suggest that animal bone-derived materials have the potential to serve as sustainable auxiliary fillers for composite applications. Although these bio-derived fillers have demonstrated promising improvements in shielding or mechanical properties, most previous studies primarily focused on utilizing calcium-based natural wastes as direct radiation shielding materials or as alternative fillers replacing conventional shielding materials [23,24,25]. In contrast, their potential role as microstructural densification modifiers for highly filled tungsten/polymer shielding composites has received little attention. In particular, systematic investigations on the influence of calcined animal-bone-derived calcium phosphate on particle packing, porosity suppression, effective density, and X-ray shielding performance remain scarce. However, from the perspective of medical shielding sheets, conventional auxiliary fillers still present limitations regarding interfacial compatibility with polymers, processability under high-filler loading conditions, shielding efficiency relative to product cost, and achieving an effective density by suppressing agglomeration and microporosity [26,27]. In particular, approaches aimed at improving the shielding performance by reducing the porosity without decreasing the tungsten content are directly associated with process stability for commercialization. Therefore, developing process-friendly auxiliary fillers is essential for practical applications in the medical environment [28].

To address these issues, this study proposes calcium phosphate (Ca–P)-based inorganic powder extracted from waste animal bones as an economical and polymer-compatible auxiliary filler that can be combined with tungsten. The mineral component of animal bones primarily comprises Ca–P-based biogenic apatite, which may exist in the form of carbonate-substituted hydroxyapatite [29]. As slaughter by-products in the meat processing industry, bovine and porcine bones are generated in large quantities and exhibit high volume and mass. Because they contain organic matter and moisture, they are susceptible to decomposition, odor generation, and hygiene-related problems, requiring additional treatment costs [30]. Despite the existence of several utilization pathways, these wastes are often consumed in low-value applications or disposed of through incineration or landfilling [31]. Converting such waste resources into inorganic functional powders for high-value applications is important from the perspectives of resource circulation and waste-treatment cost reduction. Furthermore, this approach may contribute to the design of environmentally sustainable upcycling-based materials combined with the development of lead-free medical shielding materials [32].

In this study, Ca–P was employed as a densification filler, rather than as a primary shielding material intended to replace tungsten, to compensate for effective density by suppressing agglomeration and microporosity in tungsten metal–particle composites [33]. Unlike high-atomic-number materials that primarily maximize the attenuation contribution, Ca–P functions as an auxiliary additive that actively contributes to effective attenuation from a microstructural perspective. Unlike previous studies that mainly evaluated natural waste fillers as radiation attenuating constituents, the present study focuses on the microstructural engineering effect of calcined animal-bone-derived Ca–P on highly loaded tungsten composites through densification and pore suppression. Particularly, the use of micro-scale Ca–P powder was expected to induce hybrid packing by filling the interstitial spaces between tungsten particles, reduce void formation caused by air entrapment during the mixing and calendering processes of the composite, and consequently increase the density under identical areal density or thickness conditions, thereby minimizing or improving shielding performance degradation.

The present study proposes a composite design strategy aimed at simultaneously improving processability and X-ray shielding performance, rather than relying solely on increasing the tungsten content. To achieve this, waste animal bone was calcined at temperatures ranging from 600 to 1200 °C to remove organic matter and moisture, followed by additional milling to produce micron-sized Ca–P inorganic powder. Since the calcination temperature can influence the crystallinity, surface characteristics, agglomeration behavior, and moisture absorption properties of the Ca–P powder, this study systematically investigated how different calcination conditions affect particle dispersion, void formation, and effective density within the final shielding sheets.

The shielding sheets were fabricated using a flexible polyvinyl chloride (PVC) matrix and a calendaring process to enhance their industrial applicability. PVC is a thermoplastic polymer well suited for sheet-forming processes and can provide the processability and flexibility required for highly filled metal–polymer composites. Therefore, this study evaluates the feasibility of utilizing waste animal bone-derived Ca–P as a low-cost upcycled auxiliary filler to reduce processing defects and microvoids within tungsten/PVC composite sheets. Furthermore, the study aims to verify whether the resulting improvement in effective density can contribute to enhanced X-ray shielding performance.

The purpose of this study was to improve the shielding performance of highly filled tungsten/PVC composite sheets by reducing the internal porosity and increasing the effective density by incorporating calcined animal-bone-derived Ca–P. This study differs from previous research on lead-free shielding materials because it proposes a strategy for enhancing the effective performance of highly filled tungsten composite shielding sheets using waste animal-bone-derived Ca–P as a densification filler. In addition, it provides practical evidence that may contribute to securing the process stability and performance competitiveness required for the commercialization of medical lead-free shielding materials.

## 2. Materials and Methods

### 2.1. Radiation Attenuation Theory and Density Calculation

Most radiation used for diagnostic purposes in medical institutions consists of X-rays, and X-ray shielding follows the linear attenuation law [34]. The linear attenuation equations used to calculate the degree of attenuation of the radiation energy incident on the shielding material are presented in Equations (1) and (2).

(1) $$\frac{I}{I_{0}}=e^{-\mu_{1}\chi }$$

(2) $$\mu_{1}=\left[In\left(\frac{I_{0}}{I}\right)-\mu_{2}\chi_{2}\right]/\chi_{1}$$

I = Radiation transmitted intensity;

$I_{0}$ = Radiation incident intensity;

$\mu_{1}$ = Linear attenuation coefficient of the composite material;

$\mu_{2}$ = Linear attenuation coefficient of air;

$\chi_{1}$ = Thickness of the shielding sheet;

$\chi_{2}$ = Distance between the radiation source and shielding sheet.

The linear attenuation coefficient varies depending on the thickness and density (ρ) of the constituent material, even for the same material, and the radiation incident on the shielding material can enhance the shielding efficiency by interacting with the internal constituents of the material [35]. Therefore, factors related to the density and mass of the shielding material must be considered. The mass attenuation coefficient is expressed as μ/ρ and is described in Equation (3) [36]. Accordingly, in shielding materials, the mass attenuation coefficient is influenced by the mass fraction ($\omega_{i}$) of each constituent material, as shown in Equation (4). Using the mass attenuation coefficient may contribute to achieving effective shielding performance [37].

(3) $$I=I_{0}e^{-(\frac{\mu_{1}}{\rho })\chi \rho }$$

(4) $$\frac{\mu }{\rho }=\sum_{i}\omega_{i}\left(\frac{\mu }{\rho }\right)i$$

With composite materials, variations in the mass attenuation coefficient of the shielding sheet are associated with a reduced shielding material volume (V) caused by microporosity within the sheet, which consequently decreases the density (ρ), as expressed in Equation (5) [38].

(5) $$\rho =\frac{m}{V}$$

In this study, effective density was defined as the bulk density ($\rho_{bulk}$) of the shielding sheet, including internal pores. Even under the conditions of identical composition and thickness, microporosity increases the bulk volume of the sheet, thereby reducing the density and deteriorating the X-ray shielding performance. To minimize measurement errors such as bubble adhesion, which may occur during the immersion-based density measurements of thin flexible specimens, the density of the sheet was calculated using the areal density ($m_{A}$, kg/m^2^) and thickness (t, mm). The areal density was determined from the mass (m) and area (A) of the specimen, while the thickness was obtained as the average value measured at multiple locations. The bulk density was calculated using Equation (6). The uncertainty of the bulk density was calculated by propagating the uncertainties associated with areal density and thickness measurements. Since the bulk density $\rho_{bulk}$ is defined as $\rho_{bulk}=\frac{m_{A}}{t}$, the corresponding uncertainty ($\alpha_{\rho }$) was calculated using the law of propagation of uncertainty, as expressed in Equation (7) [39]. This approach enables the estimation of changes in density according to the internal composition of the sheet, and the particle and cluster dispersion characteristics can be quantitatively compared through visual observation [40].

(6) $$\rho_{bulk}\;(g/{cm}^{3})=\frac{m_{A}\;(kg/m^{2})}{t\;(mm)}$$

(7) $$\alpha_{\rho }=\rho_{bulk}\sqrt{{\left(\frac{\alpha_{m_{A}}}{m_{A}}\right)}^{2}+{\left(\frac{\alpha_{t}}{t}\right)}^{2}}$$

### 2.2. Materials

Tungsten powder (99.9%, 19.3 g/cm^3^, <10 μm, NanGong XinDun Alloys Spraying, Nangong, China) was used as the metallic filler. To control particle size, the powder was pulverized for 5 min and subsequently dried in an oven at 70–85 °C for 24 h. Bovine bone was selected as the auxiliary filler and subjected to washing, degreasing, drying, and pulverization using a jet mill (DSML 370, Duksan). The processed bone powder was thermally treated through calcination from 600 to 1200 °C using a Muffle Furnace (Nabertherm, Model L5/11). After washing, degreasing, drying, jet milling, and calcination, the bovine bone was obtained as a micron-sized Ca–P inorganic powder. The powder was directly used as an auxiliary densification filler for melt compounding with tungsten and PVC. To investigate particle changes according to processing temperature, 600, 800, and 1200 °C were selected as the baseline calcination, intermediate, and maximum temperatures, respectively [41]. The Ca–P inorganic powders obtained at different calcination temperatures were subsequently used for sheet fabrication, and the internal structure and shielding performance of the resulting sheets were compared to determine the optimal processing conditions. The true density of Ca–P was used as an input parameter for calculating the theoretical density of the composites. In this study, a single value of 2.58 g/cm^3^ was applied regardless of calcination temperature, rather than assigning separate true density values for each calcination condition. The effects of calcination temperature were instead evaluated in terms of changes in powder composition, microstructure, and the bulk density of the final composite sheets. To eliminate the possibility of moisture absorption by the Ca–P inorganic powder prior to mixing, a pre-drying process was performed to reduce the formation of micropores during the compounding process [42].

A commercial PVC resin (DG-800T, Dagu, China) was used as the polymer matrix. PVC generally has an average molecular weight ranging from 50,000 to 110,000 and a density of approximately 1.35–1.45 g/cm^3^, which is relatively high among general-purpose plastics [43]. PVC can simultaneously satisfy the melt strength and processability requirements for the fabrication of sheets containing highly loaded metallic fillers such as tungsten [44]. Highly filled metal–particle sheets often exhibit microporosity caused by poor particle dispersion and agglomeration during the mixing process, which can reduce both the density and shielding performance [45]. Therefore, sufficient melt viscosity and mechanical strength are required to effectively retain and disperse metal particles, and PVC is a suitable material for this purpose.

The chemical composition of the calcined Ca–P powders was analyzed using X-ray fluorescence (XRF) (ZSX Primus IV, Rigaku Corporation, Japan). The oxide compositions presented in Table 1 were normalized on a loss-on-ignition (LOI)-free basis to facilitate comparison among the different calcination temperatures.

### 2.3. Fabrication of Shielding Sheets

**Figure 1:**
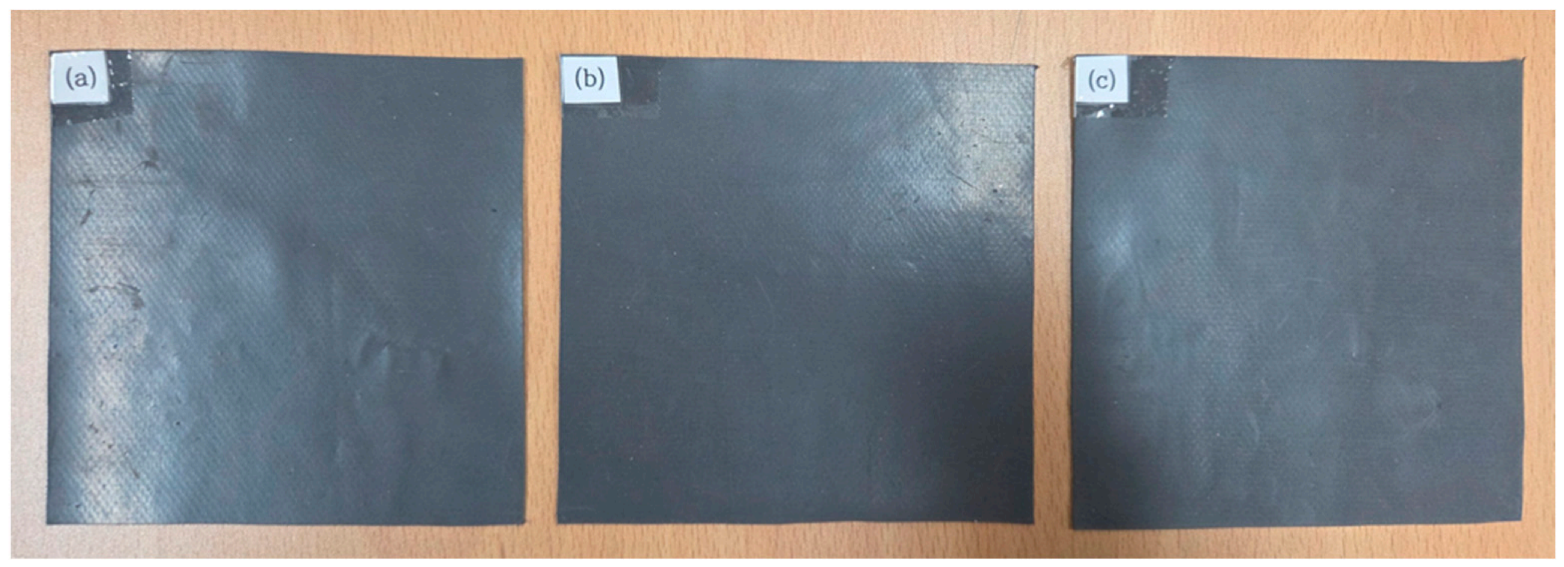
Appearance of the shielding sheets fabricated using the calendering process according to tungsten content: (**a**) 75, (**b**) 80, and (**c**) 85 wt%.

Plasticizers and stabilizers were added to the PVC base and mixed with tungsten and Ca–P for 90 min. The compounded mixture was processed using a rubber mixing mill (Two-Roll Mill, Qingdao Ouli Machine Co., Qingdao, China) at a roll temperature and speed ratio of 65–70 °C and 1:1.4, respectively. The calendering process was expected to control the sheet thickness while collapsing residual bubbles and micropores through compression and shear forces, thereby improving thickness uniformity and effective density of the sheet [46]. Because the final shielding performance is directly influenced by the tungsten loading fraction, three types of sheets were fabricated by increasing the tungsten content from 75 to 85 wt% in increments of 5 wt% (denoted as W-75, W-80, and W-85, respectively). Sheet flexibility and calendering processability were considered, and the Ca–P content was maintained at 10 wt% in all formulations. The fabricated sheets were stabilized at room temperature for >24 h prior to the performance evaluation. The final specimens used in the experiments were fabricated with dimensions of 100 mm × 100 mm × 0.25 mm, as shown in Figure 1.

To analyze the morphological structures of the shielding sheets, including their particle dispersion characteristics, thin cross-sectional specimens were prepared using a microtome (Leica RM2235, Leica, Wetzlar, Germany) and observed using field-emission scanning electron microscopy (Hitachi S-4800, Tokyo, Japan).

### 2.4. Microstructural Characterization

Accordingly, SEM cross-sectional images were quantitatively analyzed using Fiji (ImageJ; version 1.54f) to evaluate the agglomeration tendency of Ca–P and its dispersion state within the composite as a function of the processing conditions. After contrast enhancement and threshold segmentation were applied to SEM images acquired at the same magnification, connected-component analysis was performed to determine the area and equivalent circular diameter (ECD) of particle clusters. In this study, connected particle groups with an ECD ≥ 2.0 μm were defined as agglomerates, and the agglomerate area fraction and number density were calculated. However, because tungsten (W) and Ca–P could not be completely distinguished based solely on SEM image contrast, the analysis was interpreted as a morphological assessment of the overall agglomeration behavior of particulate clusters within the W/Ca–P/PVC composite rather than the agglomeration of Ca–P alone.

**Figure 2:**
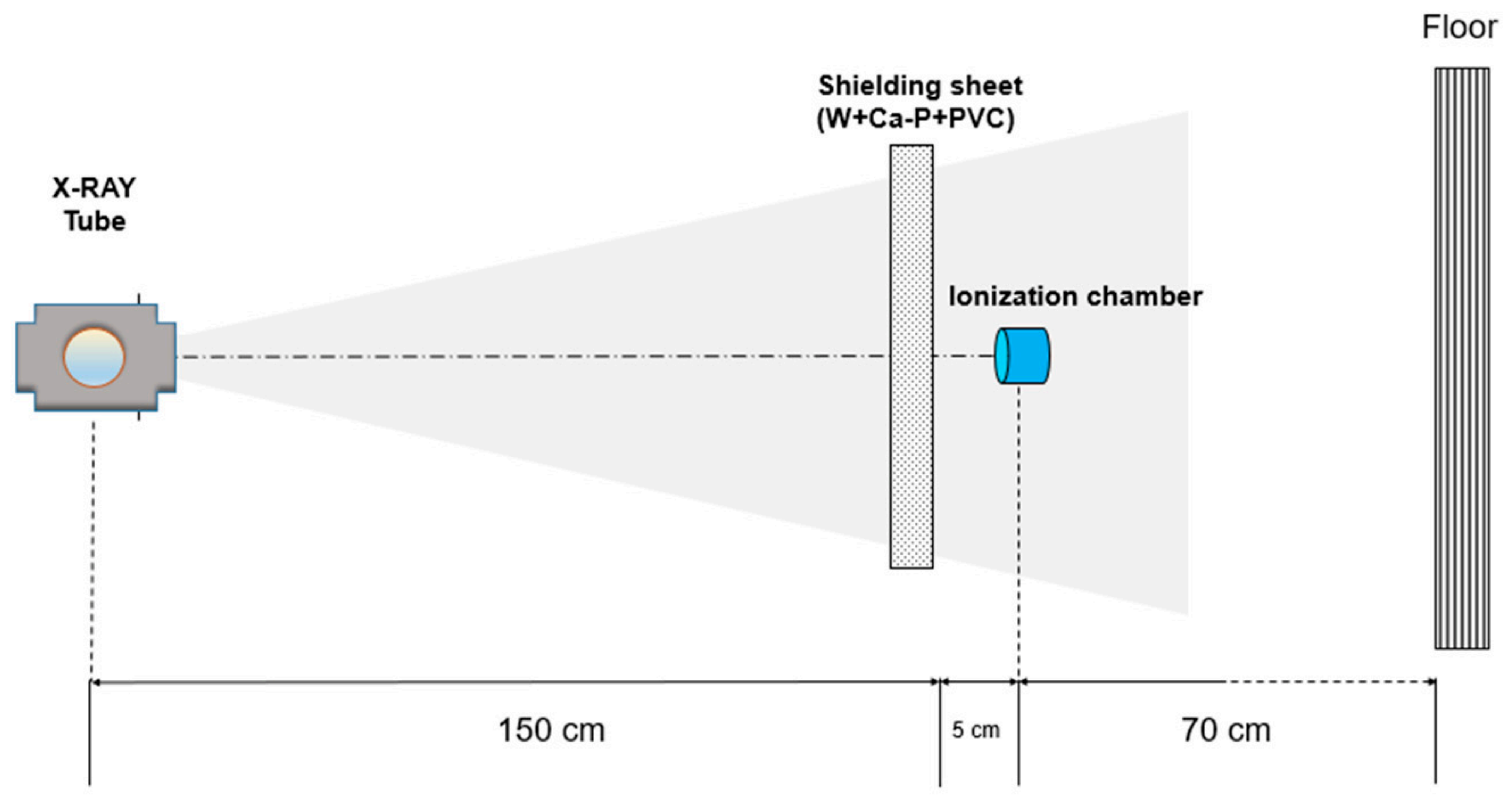
Evaluation of the radiation shielding performance of the fabricated shielding sheets. Note: W, tungsten; Ca–P, calcium phosphate; PVC, polyvinyl chloride.

The experimental setup for evaluating the shielding performance of the shielding sheets was configured under the geometric conditions shown in Figure 2.

### 2.5. X-Ray Shielding Performance Evaluation

The half-value layer (HVL) was determined by applying the attenuation law to convert the medical radiation used in the experiment into an effective energy value. Particularly, the natural logarithm was obtained on both sides of the exponential attenuation equation, while the linear attenuation coefficient μ was obtained from the slope of the resulting linear graph y=−ax. The HVL was then calculated using the relation HVL = 0.693/μ [47]. The effective energy was determined by identifying the single-energy value for which the theoretical HVL matched the experimentally obtained HVL using Hubbell’s mass attenuation coefficient tables [14]. The shielding performance was evaluated in terms of the radiation protection efficiency (RPE; %), which was calculated using Equation (8) [48].

(8) $$RPE=(1-\frac{D}{D_{0}})\times 100$$

Here, D represents the dose measured with a shielding sheet interposed between the X-ray tube and dosimeter, whereas $D_{0}$ denotes the exposure dose measured in the absence of any sheet between the X-ray tube and dosimeter. Measurements were performed in triplicate using an X-ray generator (Toshiba E7239, 150 kV, 500 mA, 1999, Kawasaki, Japan), and the mean value was used for analysis. The dose detector used was a DosiMax Plus 1 (IBA Dosimetry Corp., Herndon, VA, USA, 2019), which was verified and calibrated prior to use.

## 3. Results

The XRF results presented in Table 1 indicate that the calcined animal bone-derived Ca–P powder consisted predominantly of CaO and P_2_O_5_, confirming its suitability as a Ca–P-based inorganic filler. In particular, under the 1200 °C calcination condition, the relative mass fraction of P_2_O_5_ increased while the proportion of the others category decreased. In particular, under the 1200 °C calcination condition, the relative mass fraction of P_2_O_5_ increased while the proportion of the others category decreased, indicating that the inorganic phase became increasingly dominated by Ca–P components with increasing calcination temperature. As the calcination temperature increased, the loss on ignition (LOI) decreased, and the relative mass fractions of the primary oxides, CaO and P_2_O_5_, showed corresponding trends. In contrast, MgO and the alkali oxides (Na_2_O and K_2_O), as minor constituents, showed limited compositional variations with increasing temperature; however, a reduction in alkali content was observed for high-temperature calcination (1200 °C). However, because the compositions presented in Table 1 were normalized on an LOI-free basis, the effects of organic matter removal and volatile component loss with increasing calcination temperature should be interpreted in conjunction with separate LOI or thermogravimetric analysis (TGA) results.

Sheets (0.25 mm) were fabricated using calcined bovine-bone-derived Ca–P, which had a density of 1.82 g/cm^3^. The tungsten content was maintained at 75–85 wt%, while the Ca–P content was fixed at 10 wt%. Variations in the density of the fabricated sheets were evaluated, and the physical properties of the shielding sheets were investigated as functions of the tungsten/PVC composite composition, as summarized in Table 2. Although the density increased with increasing tungsten content, the quantitative difference was not substantial. Therefore, the PVC base material and Ca–P collectively contributed to and influenced the overall density of the composite.

**Figure 3:**
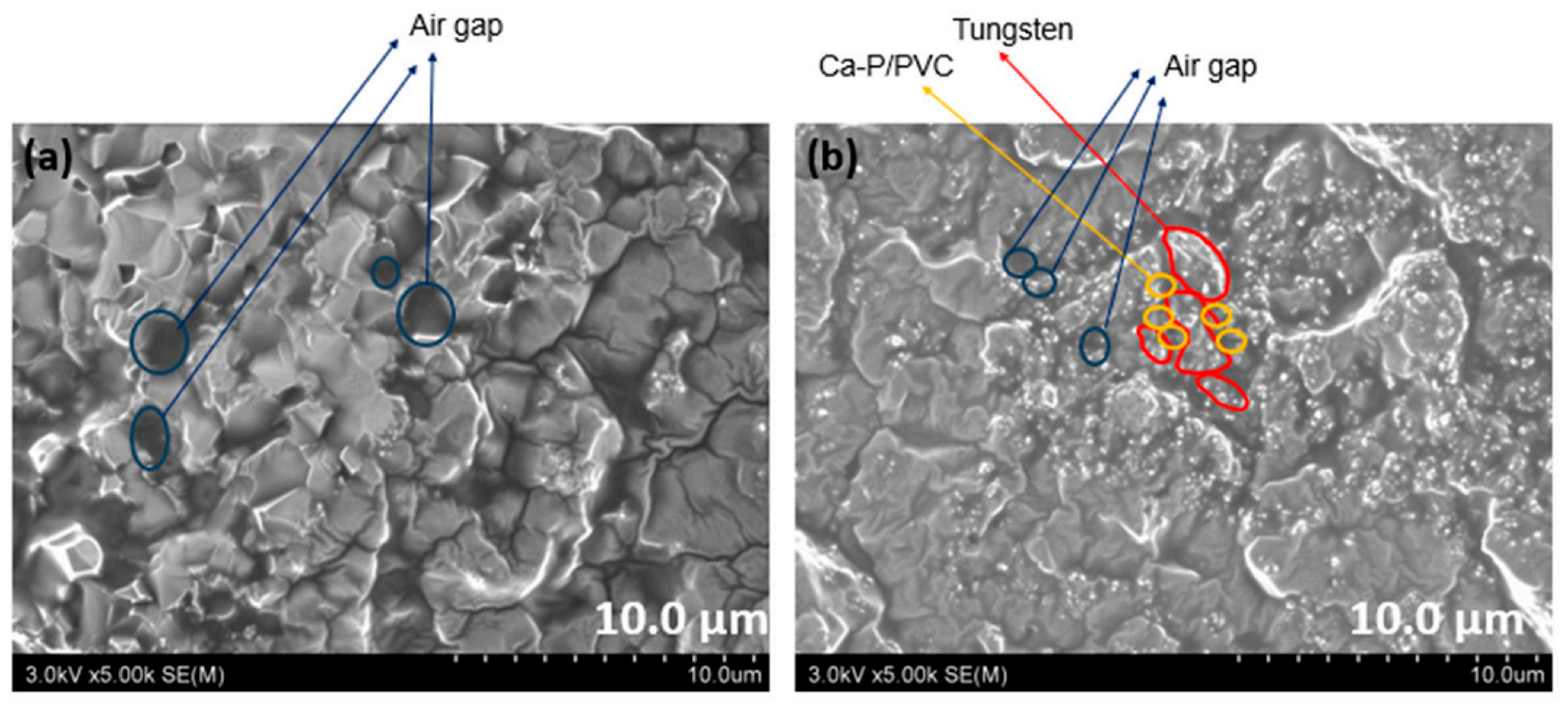
Representative SEM images showing the cross-sectional microstructure of shielding sheets fabricated (**a**) without Ca–P and (**b**) with Ca–P calcined at 600 °C. The indicated presumed tungsten-rich regions, presumed fine-particle regions, and air gaps are qualitatively interpreted based on particle morphology and are intended for microstructural comparison rather than elemental identification.

Figure 3 shows the cross-sectional microstructural changes in the W/PVC composite sheets as a function of Ca–P incorporation. In the W/PVC sheet (Figure 3a), relatively large voids and interparticle gaps were observed around the tungsten (W) particles. In contrast, the W/Ca–P/PVC sheet (Figure 3b) exhibited a noticeable reduction in void formation, suggesting a more compact internal structure. Quantitative analysis of the void area fraction will be required to further verify this trend. In addition, fine particulate regions were observed to be distributed between W particles and around the PVC matrix. It should be noted that these fine particles were interpreted as Ca–P based on SEM contrast differences, and the spatial distinction between W and Ca–P was inferred from morphological features observed in the SEM images. Because SEM–EDS elemental mapping was not performed in this study, the spatial distribution of Ca–P could not be directly confirmed. Therefore, the proposed densification mechanism was interpreted qualitatively based on SEM morphology together with the density measurements and X-ray shielding performance. For the W-85 specimen, the variation in areal density was relatively small, whereas the variation in sheet thickness was comparatively larger, resulting in a greater uncertainty in the calculated bulk density. Accordingly, the density uncertainty reported in this study was primarily attributed to local thickness non-uniformity introduced during the calendaring process.

**Figure 4:**
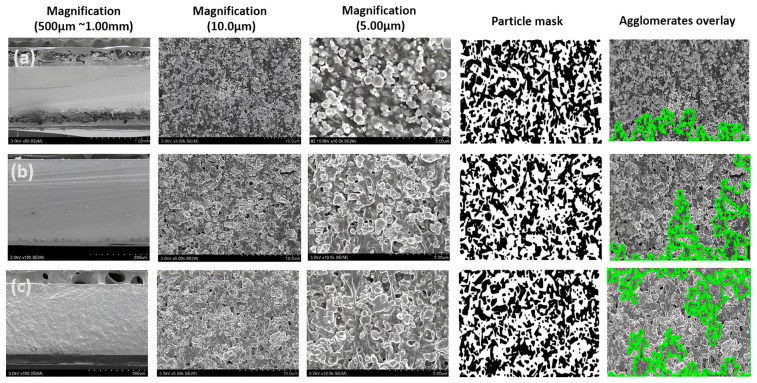
Cross-sectional images of the shielding sheets fabricated using W, Ca–P, and PVC: (**a**) 75, (**b**) 80, and (**c**) 85 wt% tungsten. The green outlines indicate the agglomerates identified by image analysis.

The cross-sectional distribution of particles in the shielding sheets as a function of tungsten content was observed by electron microscopy, as shown in Figure 4. Figure 4 presents cross-sectional images of sheets fabricated with Ca–P calcined at 600 °C, with varying tungsten content in wt%. Figure 4a shows the case in which only tungsten (75 wt%) and PVC were mixed, Figure 4b shows the cross-sectional image with tungsten (80 wt%), and Figure 4c shows the cross-sectional image with tungsten (85 wt%). Comparing Figure 4a with Figure 4b,c, the voids surrounding the tungsten particles are notably reduced, and the density is relatively improved. Furthermore, comparing Figure 4b,c, in which the tungsten content is increased, no significant difference in particle distribution was observed as a result of calcination processing at varying temperatures.

The SEM cross-sectional images were quantitatively analyzed using Fiji (ImageJ software), and the particle and cluster dispersion characteristics were compared, as summarized in Table 3. The particle/cluster area fraction remained at similar levels regardless of temperature; however, the aggregate number density and area fraction were the highest in W-85. The quantitative indicators also revealed differences consistent with those observed in the images before and after calcination. The cumulative distribution function (CDF) of the nearest-neighbor spacing (NNS) represents the cumulative distribution of distances calculated from the center of each particle and cluster to its nearest neighbor, enabling the quantification of the spatial uniformity and local clustering tendency of the particles. A leftward shift in the CDF indicates an increased proportion of particles with smaller NNS values, suggesting a higher likelihood of agglomeration, whereas a rightward shift indicates a relatively uniform dispersion.

**Figure 5:**
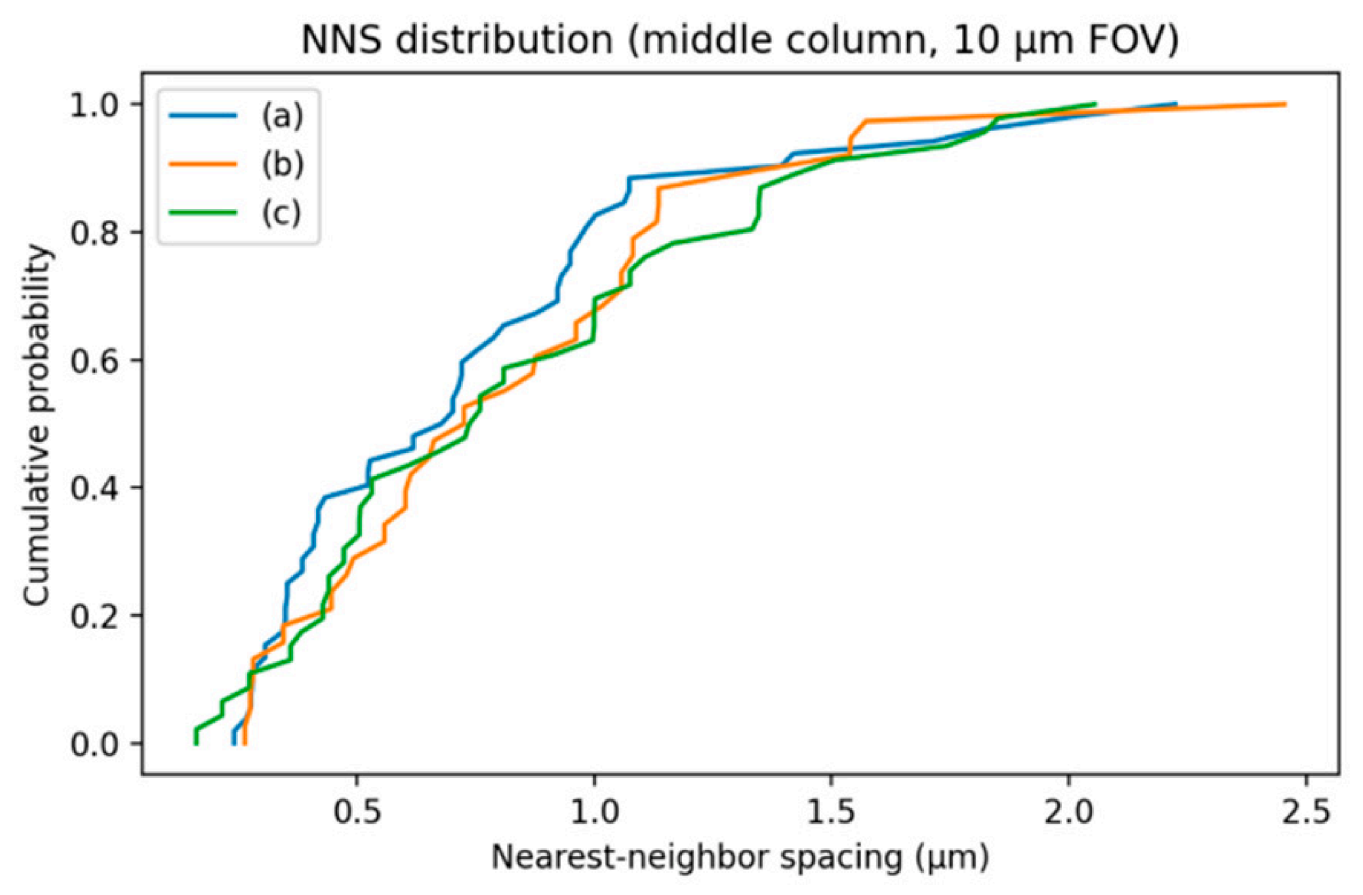
Cumulative distribution function (CDF) of nearest-neighbor spacing (NNS) derived from the scanning electron microscopy (SEM) images (10 µm field of view, FOV), (a) 75 wt% tungsten, (b) 80 wt% tungsten, and (c) 85 wt% tungsten.

Accordingly, the W-85 specimen exhibited the highest particle/cluster area fraction and agglomerate area fraction among all samples. This suggests that increasing the tungsten content led to a higher overall filler loading, thereby increasing the likelihood of localized particle-cluster formation. Meanwhile, the NNS analysis shown in Figure 5 revealed that the W-85 specimen had the largest d50 value (0.736 μm), indicating an increase in the average center-to-center distance between particles, which may adversely affect uniform particle dispersion. Therefore, the microstructure of the W-85 specimen can be interpreted as a highly filled system in which both an increase in the average interparticle spacing and an increase in localized particle agglomeration occurred simultaneously.

Table 4 presents the radiation shielding evaluation results of the fabricated sheets sintered at 800 °C. The RPE at a thickness of 0.25 mm generally decreased with increasing effective X-ray energy, and a higher tungsten content resulted in greater shielding efficiency. Following the heat treatment of Ca–P, the RPE increased across most of the energy ranges. At 23.6 keV, W-75 increased from 80.45% to 84.93% while W-80 improved from 86.24% to 89.58%. Notably, W-85 increased from 82.94% to 86.36% at mid-to-high energy (46.5 keV) and from 79.78% to 82.42% at 52.4 keV. Thus, densification conditions such as void reduction and the effective density increase resulting from heat treatment contributed to improving shielding performance.

**Figure 6:**
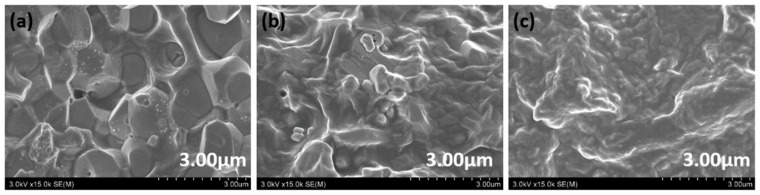
Microstructural changes in the polymer composite sheets as a function of Ca–P calcination temperature: (**a**) 600, (**b**) 800, and (**c**) 1200 °C.

SEM observations in Figure 6 revealed that the number of micropores and interparticle gaps within the composite sheets tended to decrease with increasing calcination temperature of the Ca–P filler. In particular, the specimen prepared with Ca–P calcined at 1200 °C exhibited a more continuous and compact cross-sectional morphology than that prepared at 600 °C, suggesting that calcined Ca–P may have contributed to pore reduction and improved effective density in the W/PVC composites. However, because the SEM images provide only morphological information, the observed fine-particle regions cannot be conclusively identified as Ca–P. Therefore, although Figure 6 could be interpreted as indirect evidence of Ca–P dispersion within the composite, the present study primarily considers these observations as supplementary microstructural evidence demonstrating the densification trend of the composite cross-sections with increasing Ca–P calcination temperature, rather than definitive proof of homogeneous Ca–P distribution.

As shown in Table 5, the average bulk density of the specimens before calcination increased from 11.000 g/cm^3^ for W-75 to 12.448 g/cm^3^ for W-85. However, the standard deviation of the W-85 specimen was relatively large (±1.394 g/cm^3^), resulting in partial overlap with the density ranges of the W-75 and W-80 specimens. Therefore, under the pre-calcination condition, the results are more appropriately interpreted as indicating a tendency toward increased density with increasing tungsten content, rather than conclusive evidence of a density increase. In contrast, after Ca–P calcination treatment, the bulk density increased for all compositions, with density improvements of 10.1%, 18.4%, and 22.4% observed for W-75, W-80, and W-85, respectively. Furthermore, the coefficient of variation (CV) decreased to a range of 3.22–4.05% after calcination. These results suggest that the incorporation of calcined Ca–P contributed to improved thickness uniformity and enhanced reproducibility of the effective density in highly filled composite sheets.

## 4. Discussion

Agglomeration and microporosity formation arising from high filler loading, which is commonly encountered when fabricating tungsten-based lead-free shielding sheets, can reduce the density and consequently diminish the actual X-ray shielding performance relative to the nominal composition [49]. In this study, Ca–P inorganic powder derived from waste animal bone was introduced as a supplementary tungsten shielding material, functioning as a densification filler designed to fill interparticle voids between tungsten particles and suppress porosity, thereby inducing densification of the composite. The core aim of this study was to enhance the attenuation of incident energy at equivalent thickness and areal density conditions through microstructural control during processing.

CaO and P_2_O_5_ constituted most of the Ca–P supplementary filler, and the LOI decreased with varying calcination temperature, accompanied by a shift in the relative fractions of major constituents under LOI-free normalization. This reduction in LOI was associated with the removal of residual organic components in bone meal, which is significant in terms of porosity suppression by reducing potential gas-generating sources during polymer melt mixing and nucleation sites for pore formation via moisture absorption [50,51]. The heat-treatment temperature altered the structure and surface characteristics of Ca–P, which is consistent with previous studies [52]. Bone-derived apatite may undergo a sharp increase in porosity at 600 °C due to organic combustion and moisture release, while at higher temperatures, porosity decreases alongside increased crystallinity and grain growth, with structural changes observed at 600–800 °C [53]. This study selected calcination temperatures of 600, 800, and 1200 °C to compare the process compatibility of Ca–P fillers. The results indicated that crystallization and surface state changes induced by heat treatment, rather than compositional ratios alone, influenced pore formation during mixing and calendering.

A tendency toward porosity suppression by applying Ca–P was confirmed through microstructural observation. Compared to those without Ca–P, sheets incorporating heat-treated Ca–P exhibited fewer voids around the tungsten particles and a relatively denser microstructure. Although the particle and cluster area fractions varied within a range showing no substantial difference with the calcination temperature, the aggregate area fraction simultaneously increased under high-temperature conditions. CDF analysis based on NNS also suggested a relatively uniform spatial dispersion [54].

Therefore, the aggregate area fraction and spatial uniformity may not always move in the same direction. Even if high-temperature calcined Ca–P is accompanied by some degree of agglomeration, the possibility that this ultimately results in homogenization of the overall interparticle spacing distribution and reduction in porosity must be considered. Because Ca–P was introduced as a low-content densification modifier rather than as a replacement for tungsten, localized accumulation of Ca–P does not necessarily create tungsten-depleted regions. Instead, the primary role of Ca–P is to occupy interparticle voids, thereby improving the effective density of the composite while maintaining the overall tungsten content. Moreover, the factor governing the performance of highly loaded composites is not merely the presence or absence of agglomerates but also the effective density created by the interplay of agglomerates, pores, and interparticle gaps. The density results obtained in this study support this interpretation.

The density of the shielding sheets increased from 11.000 to 12.448 g/cm^3^ with increasing tungsten content. However, the relatively large deviations suggest that, in addition to the compositional effect, microporosity and particle dispersion developed during the mixing and calendering processes also influenced the density. Notably, a significant increase in density was observed after the incorporation of calcined Ca–P powder compared with the non-calcined condition. These findings suggest that calcined Ca–P contributed to improving the uniformity and reproducibility of microstructural densification, particularly under high tungsten-loading conditions. Thus, the calcination conditions of Ca–P can function in a direction that reduces the effective density loss.

The shielding performance also exhibited a tendency consistent with the density variation. The RPE showed a typical decreasing trend with increasing effective X-ray energy and an increasing trend with increasing tungsten content. In particular, the RPE increased across most energy ranges following the application of calcined Ca–P, thereby highlighting the importance of the mass attenuation coefficient and density reduction due to porosity. Consequently, shielding performance is not simply determined by the atomic number and content alone but is directly linked to the bulk density reduced by internal voids within the composite.

Previous studies employing bio-derived fillers have primarily focused on utilizing agricultural wastes or natural calcium-containing materials as direct shielding fillers or reinforcing constituents of polymer composites [23,25]. In contrast, the present study did not aim to replace tungsten with bio-derived material. Instead, calcined animal bone-derived Ca–P functioned as a densification modifier that improved particle packing by filling interstitial spaces between tungsten particles, thereby suppressing microporosity, increasing effective density, and ultimately enhancing X-ray shielding performance. This microstructural engineering strategy fundamentally differs from previous bio-filler approaches that mainly relied on direct replacement of conventional shielding fillers.

Previous studies on tungsten-based flexible shielding composites have generally improved shielding performance by increasing the tungsten loading or incorporating high-atomic-number secondary fillers such as Bi_2_O_3_, BaSO_4_, and WO_3_ [18,26,27]. In contrast, the present study adopted a different strategy in which calcined animal bone-derived Ca–P was introduced as a low-content densification modifier rather than as a primary shielding filler. Although the shielding improvement observed in this study was modest, the results demonstrate that enhancing the effective density through suppression of microporosity can improve shielding performance without increasing the tungsten content. These findings suggest that optimization of the composite microstructure, rather than simply increasing the concentration of high-Z fillers, represents a feasible strategy for improving the shielding efficiency of flexible medical radiation shielding materials.

The approach proposed in this study has practical significance for developing eco-friendly shielding materials for low-dose medical radiation environments. Scatter radiation protection in diagnostic and interventional procedures involves prolonged wear and handling, rendering high-energy shielding, processability, sheet weight, and uniformity important. The Ca–P supplementary filler satisfies both environmental and manufacturing compatibility requirements because it is derived from waste resources with high raw material accessibility, and organic matter removal and stability can be achieved through the calcination process [55]. The shielding performance improvement based on the effective density enhancement represents a strategy that can elevate performance without simply increasing the tungsten content. This approach offers the potential for improving the economic efficiency and wearability by reducing tungsten usage or sheet thickness under equivalent performance standards. Furthermore, sheets fabricated based on melt-mixing and calendering processes may benefit from calendering pressing and shear action, which can collapse residual air bubbles and microporosity, thereby contributing to improved thickness uniformity and effective density and indicating scalability [46].

A limitation of this study is that the tungsten content and calcination temperature of the Ca–P shielding auxiliary were simultaneously varied in the experimental design, rendering it difficult to isolate the independent effects of the calcination temperature. In addition, physicochemical characterization of the calcined Ca–P powder, including X-ray diffraction (XRD), particle size distribution, and specific surface area measurements, was not performed in the present study. Therefore, the discussion regarding the influence of calcination on the physicochemical properties of Ca–P was based on XRF, LOI, SEM observations, density measurements, X-ray shielding performance, and previous literature. Furthermore, quantitative characterization of porosity, including the void area fraction, average pore size, and pore size distribution, was not performed in the present study. Future studies should incorporate quantitative porosity analysis to establish a more direct relationship between porosity reduction, effective density, and X-ray shielding performance, and to further clarify the calcination-induced densification mechanism of the composite. Future studies should employ a full factorial design with fixed tungsten and Ca-P contents to strengthen causal inference. In addition, practical evaluations relevant to wearable shielding applications, including flexibility, bending fatigue, mechanical strength, aging behavior, thermal stability, and comparisons with commercial shielding materials, should be conducted to further validate the practical applicability of the proposed composite sheets under long-term service conditions.

## 5. Conclusions

By applying calcination-treated Ca–P derived from waste animal bone to tungsten and highly loaded PVC shielding sheets, the X-ray shielding efficiency was improved by >3%, accompanied by an increase in density attributable to microporosity reduction. In particular, the RPE exhibited a proportional correlation with density, indicating that Ca–P can elevate the effective shielding performance as a densification filler. Furthermore, calcined Ca–P demonstrated a shielding performance improvement of >4.2% compared with its non-calcined counterpart. If Ca–P is utilized as a supplementary material in lead-free eco-friendly shielding materials, it will contribute to securing the shielding performance competitiveness of medical institution shielding devices based on process stability and reproducibility.

## Figures and Tables

**Table 1:** Major oxide composition of animal bone-derived Ca–P powders as a function of calcination temperature (XRF, LOI-free normalized wt%).

Component (wt%, LOI-Free Normalized)	600 °C	800 °C	1200 °C
CaO	52.52	46.51	46.92
P_2_O_5_	41.12	45.12	48.81
SiO_2_	0.53	1.06	0.68
MgO	0.10	1.22	0.01
SrO	1.06	1.02	0.75
Na_2_O + K_2_O	0.46	0.62	0.31
Others	3.21	4.45	2.52

Values were normalized on an LOI-free basis. “Others” includes minor oxide components not individually listed in the table.

**Table 2:** Characteristics of the shielding sheets as a function of tungsten content.

Composition Ratio of the Sheet (vol%)	Weight (kg/m^2^)	Thickness (mm)	Density (g/cm^3^)
W(75)/PVC(15)/Ca–P(10)	2.750 ± 0.021	0.250 ± 0.011	11.000 ± 0.491
W(80)/PVC(10)/Ca–P(10)	2.982 ± 0.003	0.250 ± 0.012	11.928 ± 0.573
W(85)/PVC(5)/Ca–P(10)	3.112 ± 0.001	0.250 ± 0.028	12.448 ± 1.394

**Table 3:** Quantitative style analysis for tungsten particle affinity according to tungsten contents (wt%).

Tungsten Content	Area Fraction of Particles/Clusters (%)	Area Fraction of Agglomerates (%)
75 wt%	50.21	40.12
80 wt%	54.04	45.09
85 wt%	55.09	47.17

**Table 4:** Comparative evaluation of the shielding performance of sheets fabricated with W, Ca–P, and PVC.

Effective X-Ray Energy (keV)	RPE (%) (Thickness: 0.25 mm)								
	Ca–P Non-Containing Shielding Sheet			Before Calcination of Ca–P Filler			After Calcination of Ca–P Filler		
	W-75	W-80	W-85	W-75	W-80	W-85	W-75	W-80	W-85
23.6	78.12	85.10	92.52	80.45	86.24	94.12	84.93	89.58	94.71
25.2	76.65	83.01	83.65	78.87	85.47	88.12	81.47	87.78	91.98
31.2	70.91	78.89	81.10	75.94	82.55	86.12	80.47	84.41	88.11
46.5	68.01	77.11	79.41	71.87	80.21	82.94	72.86	82.12	86.36
52.4	62.50	72.33	75.74	64.12	76.32	79.78	65.74	78.11	82.42

**Table 5:** Uncertainty interpretation of bulk density before and after Ca–P calcination.

Condition	Sample	Density (g/cm^3^)	Range Based on ± Value (g/cm^3^)	CV (%)
Before calcination	w-75	11.000 ± 0.491	10.509–11.471	4.46
	w-80	11.928 ± 0.573	11.355–12.501	4.80
	w-85	12.448 ± 1.394	11.054–13.842	11.20
After calcination	w-75	12.111 ± 0.491	11.620–12.602	4.05
	w-80	14.121 ± 0.491	13.630–14.612	3.48
	w-85	15.241 ± 0.491	14.750–15.732	3.22

CV: Coefficient of Variation.

## Data Availability

All data generated during this study are included in this manuscript.

## Abbreviations

The following abbreviations are used in this manuscript:

Ca–P	calcium phosphate
PVC	polyvinyl chloride
SEM	scanning electron microscopy
ECD	equivalent circular diameter
HVL	half-value layer
RPE	radiation protection efficiency
LOI	loss of ignition
CDF	cumulative distribution function
NNS	nearest-neighbor spacing

## References

[1] Shbeer A. (2024). Radiation in the intensive care units: A review of staff knowledge, practices, and radiation exposure. J. Radiat. Res. Appl. Sci..

[2] Zeng C., Kang Q., Duan Z., Qin B., Feng X., Lu H., Lin Y. (2023). Development of polymer composites in radiation shielding applications: A review. J. Inorg. Organomet. Polym. Mater..

[3] Wu S., Bao J., Gao Y., Hu W., Lu Z. (2024). Review on flexible radiation-protective clothing materials. J. Mater. Sci..

[4] Maghrabi H.A., Vijayan A., Deb P., Wang L. (2016). Bismuth oxide-coated fabrics for X-ray shielding. Text. Res. J..

[5] Meisinger Q.C., Stahl C.M., Andre M.P., Kinney T.B., Newton I.G. (2016). Radiation protection for the fluoroscopy operator and staff. AJR Am. J. Roentgenol..

[6] Wu S., Zhang W., Yang Y. (2024). Progress in flexible and wearable lead-free polymer composites for radiation protection. Polymers.

[7] Safari A., Rafie P., Taeb S., Najafi M., Mortazavi S.M.J. (2024). Development of lead-free materials for radiation shielding in medical settings: A review. J. Biomed. Phys. Eng..

[8] Li X.P., Yao H.S., Zhao Y., Yuan B., Zhai J., Li L., Li H., Li X. (2025). Polymer-based nuclear radiation shielding materials: State-of-the-art and emerging trends for engineering applications. Front. Mater..

[9] AbuAlRoos N.J., Amin N.A.B., Zainon R. (2019). Conventional and new lead-free radiation shielding materials for radiation protection in nuclear medicine: A review. Radiat. Phys. Chem..

[10] Monaco M.G.L., Carta A., Tamhid T., Porru S. (2020). Anti-X apron wearing and musculoskeletal problems among healthcare workers: A systematic scoping review. Int. J. Environ. Res. Public Health.

[11] Kim S.C. (2026). Radiation Attenuation Performance of Highly Filled Tungsten/TPU Composites via Anchor–Chain Dispersant-Based Interfacial Design. Polymers.

[12] Alanazi S., Alshadokhi S., Alosime E., Almurayshid M., Alsuhybani M., Marashdeh M. (2026). Mechanical, Thermal and X-Ray Shielding Properties of Lead-Free Composites of HDPE Filled with Metal-Based Powders. Polymers.

[13] Abualroos N.J., Idris M.I., Ibrahim H., Kamaruzaman M.I., Zainon R. (2024). Physical, mechanical, and microstructural characterisation of tungsten carbide-based polymeric composites for radiation shielding application. Sci. Rep..

[14] Hubbell J.H., Seltzer S.M. (1995). Tables of X-ray Mass Attenuation Coefficients and Mass Energy-Absorption Coefficients from 1 keV to 20 MeV for Elements Z = 1 to 92 and 48 Additional Substances of Dosimetric Interest.

[15] Jayakumar S., Saravanan T., Philip J. (2023). A review on polymer nanocomposites as lead-free materials for diagnostic X-ray shielding: Recent advances, challenges and future perspectives. Hybrid Adv..

[16] Hubbell J.H. (2006). Review and history of photon cross section calculations. Phys. Med. Biol..

[17] Abunahel B.M., Ramli R.M., Quffa K.M., Azman N.Z.N. (2018). Effect of nanofibrous porosity on the X-ray attenuation properties of electrospun n-Bi2O3/epoxy–polyvinyl alcohol (PVA) nanofiber mats. Appl. Phys. A.

[18] Abdolahzadeh T., Morshedian J., Ahmadi S. (2023). Novel polyethylene/tungsten oxide/bismuth trioxide/barium sulfate/graphene oxide nanocomposites for shielding against X-ray radiations. Int. J. Radiat. Res..

[19] Neuhaus L., Ioannou O., Overend M. (2025). Bulk fillers from food waste for polymeric bio-composites: The influence of filler type, particle size and volume ratio on furan-matrix composites. Constr. Build. Mater..

[20] Mahmoud A.A., El-Sayed A.A., Fathy I.N., Fawzy S., Alturki M., Elfakharany M.E., Abouelnour M.A., Mahmoud K.A., Dahish H.A., Eltalawy S.M. (2025). Evaluation of rice husk biochar influence as a partial cement replacement material on the physical, mechanical, microstructural, and radiation shielding properties of ordinary concrete. Sci. Rep..

[21] Olivieri F., Avolio R., Gentile G., Mazzone A., Rizzi R., Nacucchi M., Castaldo R., Errico M.E., Giannini C., Ambrosio L. (2024). High filler content acrylonitrile-butadiene-styrene composites containing tungsten and bismuth oxides for effective lead-free X-ray radiation shielding. Polym. Compos..

[22] Gilys L., Griškonis E., Griškevičius P., Adlienė D. (2022). Lead free multilayered polymer composites for radiation shielding. Polymers.

[23] Tochaikul G., Tanadchangsaeng N., Panaksri A., Moonkum N. (2025). Enhancing radiation shielding capabilities with epoxy-resin composites reinforced with coral-derived calcium carbonate fillers. Polymers.

[24] Tochaikul G., Moonkum N. (2024). Development of sustainable X-ray shielding materials: An innovative approach using epoxy composite with crab shell, eggshell, and bone waste. Radiat. Phys. Chem..

[25] Pekdemir M.E., Pekdemir S.S., Yılmaz D., Onay H., Nazem Qader I. (2025). Snail Shell-Reinforced Waste-Based Polymer Composites for Radiation Shielding and Anti-Reflective Applications. Polymers.

[26] Prabhu S., Bubbly S.G., Gudennavar S.B. (2023). X-ray and γ-ray shielding efficiency of polymer composites: Choice of fillers, effect of loading and filler size, photon energy and multifunctionality. Polym. Rev..

[27] Chang Q., Guo S., Zhang X. (2023). Radiation shielding polymer composites: Ray-interaction mechanism, structural design, manufacture and biomedical applications. Mater. Des..

[28] Wu Y., Cao Y., Wu Y., Li D. (2020). Mechanical properties and gamma-ray shielding performance of 3D-printed poly-ether-ether-ketone/tungsten composites. Materials.

[29] Kono T., Sakae T., Nakada H., Kaneda T., Okada H. (2022). Confusion between carbonate apatite and biological apatite (carbonated hydroxyapatite) in bone and teeth. Minerals.

[30] Al-Gheethi A., Ma N.L., Rupani P.F., Sultana N., Yaakob M.A., Mohamed R.M.S.R., Soon C.F. (2023). Biowastes of slaughterhouses and wet markets: An overview of waste management for disease prevention. Environ. Sci. Pollut. Res..

[31] Pereira A.C., Dos Santos J.R., Da Silva J.V.F. (2025). Animal Bone Processing for a Circular Bioeconomy: Methods, Products, Applications, and Policy (2020–2025 Review). Rev. Multidiscip. Nordeste Min..

[32] Hossain R., Ghose A., Sahajwalla V. (2025). Circular economy of the materials in the healthcare industry: Opportunities and challenges. Resour. Conserv. Recycl..

[33] Yerou A., Khatir O., Bouziane M.M., Elmeguenni M., Khedr M., Järvenpää A. (2025). Eco-friendly reinforcement of dental composites with poly (methyl methacrylate) and hydroxyapatite synthesized from eggshell waste. Dent. Mater..

[34] Khan F.M., Gibbons J.P. (2014). Khan’s The Physics of Radiation Therapy.

[35] Seenappa L., Manjunatha H.C., Chandrika B.M., Chikka H. (2017). A study of shielding properties of X-ray and gamma in barium compounds. J. Radiat. Prot. Res..

[36] More C.V., Alsayed Z., Badawi M.S., Thabet A.A., Pawar P.P. (2021). Polymeric composite materials for radiation shielding: A review. Environ. Chem. Lett..

[37] Obeid A., El Balaa H., El Samad O., Awad R., Badawi M.S. (2022). Attenuation parameters of HDPE filled with different nano-size and bulk WO3 for X-ray shielding applications. Eur. Phys. J. Plus.

[38] Ortiz Sánchez C., Medina Del Barrio J.J., Fernández Romero G., Martínez Á.Y., Losada P.R., Arriaga Arellano L.A. (2025). Characterization of Novel Composite Materials with Radiation Shielding Properties for Electronic Encapsulation. Materials.

[39] Chen S., Bourham M., Rabiei A. (2014). Novel light-weight materials for shielding gamma ray. Radiat. Phys. Chem..

[40] Toto E., Lambertini L., Laurenzi S., Santonicola M.G. (2024). Recent advances and challenges in polymer-based materials for space radiation shielding. Polymers.

[41] Pang Y.X., Bao X. (2003). Influence of temperature, ripening time and calcination on the morphology and crystallinity of hydroxyapatite nanoparticles. J. Eur. Ceram. Soc..

[42] Chen C., Zhang W., Li Y., Gu L., Luo Y., Qin M., Zhang J., He Z., Wang H., Zheng Y. (2023). The Influence of Moisture Absorption-Drying of Composite Materials on the Bonding Performance of the Joints. Macromol. Mater. Eng..

[43] Dong K., Xue C., Nan H., Huang W., Zhong H., Liu G., Yang G., Xu S., Zhang J. (2024). Synthesized the cold-resistant plasticizer epoxidized 1, 6-hexanediol oleate and effects on the properties of polyvinyl chloride. Polym. Bull..

[44] Khorami M.T., Salehi Z. (2025). Characterization of the light and flexible nonlead aprons as an alternative to Pb–PVC. J. Appl. Clin. Med. Phys..

[45] Sankhla A.M., Patel K.M., Makhesana M.A., Giasin K., Pimenov D.Y., Wojciechowski S., Khanna N. (2022). Effect of mixing method and particle size on hardness and compressive strength of aluminium based metal matrix composite prepared through powder metallurgy route. J. Mater. Res. Technol..

[46] Kim S.C. (2023). Metal Particle Pencil Beam Spray-Coating Method for High-Density Polymer–Resin Composites: Evaluation of Radiation-Shielding Sheet Properties. Materials.

[47] Ganapol B.D. (2011). An analytical multigroup benchmark for (n, γ) and (n, n′, γ) verification of diffusion theory algorithms. Ann. Nucl. Energy.

[48] Chaparro-Garnica C.Y., Bailón-García E., Davó-Quiñonero A., Da Costa P., Lozano-Castelló D., Bueno-López A. (2021). High performance tunable catalysts prepared by using 3D printing. Materials.

[49] Kim S.C., Cho S.H. (2019). Analysis of the correlation between shielding material blending characteristics and porosity for radiation shielding films. Appl. Sci..

[50] Gao C., Peng S., Feng P., Shuai C. (2017). Bone biomaterials and interactions with stem cells. Bone Res..

[51] Sampath U.G.T., Ching Y.C., Chuah C.H., Sabariah J.J., Lin P.C. (2016). Fabrication of porous materials from natural/synthetic biopolymers and their composites. Materials.

[52] Tresnasari D.R., Juwono A.L., Soejoko D.S., Mulyaningsih N.N. (2019). Effects of Heat Treatment Temperature and Atmosphere on the Morphology and Structure of Calcium Phosphate. IOP Conf. Ser. Mater. Sci. Eng..

[53] Destainville A., Champion E., Bernache-Assollant D., Laborde E. (2003). Synthesis, characterization and thermal behavior of apatitic tricalcium phosphate. Mater. Chem. Phys..

[54] Clark P.J., Evans F.C. (1954). Distance to nearest neighbor as a measure of spatial relationships in populations. Ecology.

[55] Hart A., Ebiundu K., Peretomode E., Onyeaka H., Nwabor O.F., Obileke K. (2022). Value-added materials recovered from waste bone biomass: Technologies and applications. RSC Adv..

